# Detailed Analyses of Zika Virus Tropism in Culex quinquefasciatus Reveal Systemic Refractoriness

**DOI:** 10.1128/mBio.01765-20

**Published:** 2020-08-18

**Authors:** Hannah J. MacLeod, George Dimopoulos

**Affiliations:** a W. Harry Feinstone Department of Molecular Microbiology and Immunology, Bloomberg School of Public Health, Johns Hopkins University, Baltimore, Maryland, USA; EPFL

**Keywords:** *Aedes aegypti*, *Culex quinquefasciatus*, Zika virus, vector competence

## Abstract

The role of Culex quinquefasciatus in Zika virus transmission has been debated since the epidemic of Zika occurred in the Americas in 2015 to 2016. The majority of studies have found no evidence that *C. quinquefasciatus* or other *Culex* species are competent vectors of Zika virus, and the few studies that have proposed Zika vector status for *C. quinquefasciatus* have relied predominantly on quantitative real-time PCR (qRT-PCR) for viral detection. We assessed the infectious range of pre- and post-epidemic Zika virus isolates in order to classify mosquito samples based on titer infectiousness and demonstrated that two strains of *C. quinquefasciatus*, including one previously found to be competent, are highly resistant to infection with these Zika isolates compared to Aedes aegypti and are not competent for virus transmission. Further dissection of the dynamics of Zika exposure in both A. aegypti and *C. quinquefasciatus* revealed that while virus transmission by *C. quinquefasciatus* is blocked at the levels of the midgut and salivary glands, viral RNA persists in these tissues for prolonged periods post-exposure. We assessed Zika entry dynamics in both *Aedes* and *Culex* cells, and our results suggest that Zika virus infection in *Culex* cells may be blocked downstream of cell entry. These findings strongly suggest that *C. quinquefasciatus* is not a vector of Zika virus and additionally inform the use of qRT-PCR in vector competence assays as well as our understanding of barriers to arbovirus infection in non-susceptible mosquito species.

## Abstract (precis)

Understanding which mosquito species transmit an emerging arbovirus is critical to effective vector control. During the Zika virus epidemic in 2015 to 2016, *Aedes* mosquitoes were confirmed as vectors. However, studies addressing the vector status of Culex quinquefasciatus mosquitoes presented conflicting evidence and remain an outstanding source of confusion in the field. Here, we established a robust cell-based assay to identify infectious titers of Zika virus and assessed the virus titers in *C. quinquefasciatus* by quantitative real-time PCR (qRT-PCR). We found that while low levels of virus were detected in *C. quinquefasciatus*, these titers did not correspond to infectious virus, and these mosquitoes did not transmit virus in the saliva. We also present evidence that the virus may enter *Culex* cells before infection is disrupted. Our findings are important for future studies incriminating vector species using qRT-PCR for virus detection and offer new information on how virus transmission is blocked by mosquitoes.

## INTRODUCTION

The threat of emerging and reemerging arboviruses has long been recognized, but the Zika virus (ZIKV) epidemic that took place in the Americas from 2015 to 2016 (1) has called new attention to the burden of diseases caused by these pathogens. After its introduction in Brazil in 2014, ZIKV spread rapidly through the naive American population, causing hundreds of thousands of cases in Brazil alone, and spreading to 28 countries by early 2016 (2, 3). Several hypotheses were posed regarding the unique spread and pathogenesis of ZIKV in the Americas, including a lack of preexisting immunity in the American population, virus mutation and adaptation during its geographic spread, and increased ZIKV transmission due to a more susceptible and/or more abundant mosquito vector population (4–6).

A mosquito that transmits an arbovirus, or “competent vector,” acquires infectious virions when a female mosquito takes a blood meal from an infected host. From there, the virus binds and enters midgut epithelial cells, where it replicates and produces infectious virus particles that disseminate into the hemocoel of the mosquito. To be transmitted, the virus must bind and enter the epithelial cells of the salivary glands, where another cycle of virus replication occurs before virions are inoculated in the saliva during subsequent blood feeding. In resistant or refractory mosquito species or strains, virus transmission is severely reduced or prevented by “barriers” that exist at the levels of infection and dissemination for each transmission-relevant tissue (7, 8). The barriers that reduce viral infection intensity in competent vectors have been intensely investigated (9–11). However, less emphasis has been placed on understanding these interactions in non-competent mosquito species. Studies investigating these types of host-pathogen interactions have greatly informed our understanding of factors critical to vector competence (12–17).

Understanding which mosquito species are transmitting an emerging vector-borne disease in a new region is necessary for effective vector control, which remains the primary method of preventing vector-borne diseases (18, 19). While Aedes aegypti and Aedes albopictus mosquitoes have been confirmed as vectors of ZIKV in the American epidemic (20–24), the potential for *Culex* species mosquitoes, and particularly, Culex quinquefasciatus, to be involved in transmission was suggested early on as an explanation for the rapid spread of ZIKV in this region (25, 26), since these mosquitoes vector other flaviviruses and can be more abundant than *Aedes* species in Brazil (27). While some studies have suggested that different strains of *C. quinquefasciatus* are competent vectors for different ZIKV isolates by using a quantitative real-time PCR (qRT-PCR)-based approach (28–30), and one study identified plaque-forming virions in the saliva of these mosquitoes (31), the preponderance of studies using qRT-PCR and infectious virus assays have found *C. quinquefasciatus* to be noncompetent for ZIKV transmission (see Table S1 in the supplemental material). The discrepancy regarding the vector status of *C. quinquefasciatus* for ZIKV, and the potential biological and technical explanations for the divergent findings of studies on this topic, remains an outstanding source of confusion in the vector biology field.

10.1128/mBio.01765-20.9 TABLE S1 All published studies in which the competence of *Culex* species mosquitoes for ZIKV are listed with information about the mosquito strains, virus isolates, and tissues tested, as well as the infectious assay and qRT-PCR results, when available. In cases where the GenBank accession number of the virus isolate was not included in the publication, the strain name is included in parentheses. Results from studies finding evidence of ZIKV transmission by *Culex* mosquitoes are italicized. Download Table S1, DOCX file, 0.1 MB. Copyright © 2020 MacLeod and Dimopoulos. 2020 MacLeod and Dimopoulos This content is distributed under the terms of the Creative Commons Attribution 4.0 International license.

Central to determining whether a mosquito species is competent for a given arbovirus is the methodology used to detect an arbovirus within mosquito tissues. Detection of infectious virions is the gold standard for virus detection and quantification; however, cultured-based methods are often not feasible, and faster, more sensitive quantification techniques are valuable (32–34). qRT-PCR allows for the rapid and sensitive detection of viral genomic material (35–37) but cannot be used to draw conclusions about virus infectivity without additional assays. Furthermore, using qRT-PCR to determine vector competence can be complicated by the fact that small amounts of viral nucleic acids may be detected but do not indicate virus replication or transmissible titers (38–40). Correlating levels of viral nucleic acids to infectious doses of virus can improve the utility and validity of qRT-PCR for incriminating mosquito species as disease vectors.

In this study, we examined the difference in ZIKV vector competence between A. aegypti and *C. quinquefasciatus* mosquitoes in order to identify potentially differing virus dynamics between these species and discover barriers to ZIKV infection and transmission. We first correlated virus titers measured by qRT-PCR to the ZIKV inoculum required to establish infection in a highly susceptible *in vitro* assay in order to categorize mosquito samples based on whether they were likely to contain infectious virus. Based on this approach, we found both a laboratory strain of *C. quinquefasciatus* and a more recently collected strain from Hainan Province, China, to be highly resistant to ZIKV infection and refractory to ZIKV transmission compared to that of laboratory A. aegypti, even though the Hainan *C. quinquefasciatus* strain was found highly competent by a previous study (28). We further identified systemic infection barriers to ZIKV infection in laboratory *C. quinquefasciatus* but also observed persistence of viral RNA in tissues in the absence of virus replication. Our data suggest that ZIKV invades *Culex* mosquito cells and may be blocked downstream of cell entry. These findings combine molecular and culture-based methods to further our understanding of ZIKV dynamics and infection barriers in non-competent mosquito species.

## RESULTS

### Sample ZIKV titers can be categorized based on likelihood of infectiousness.

**FIG 1:**
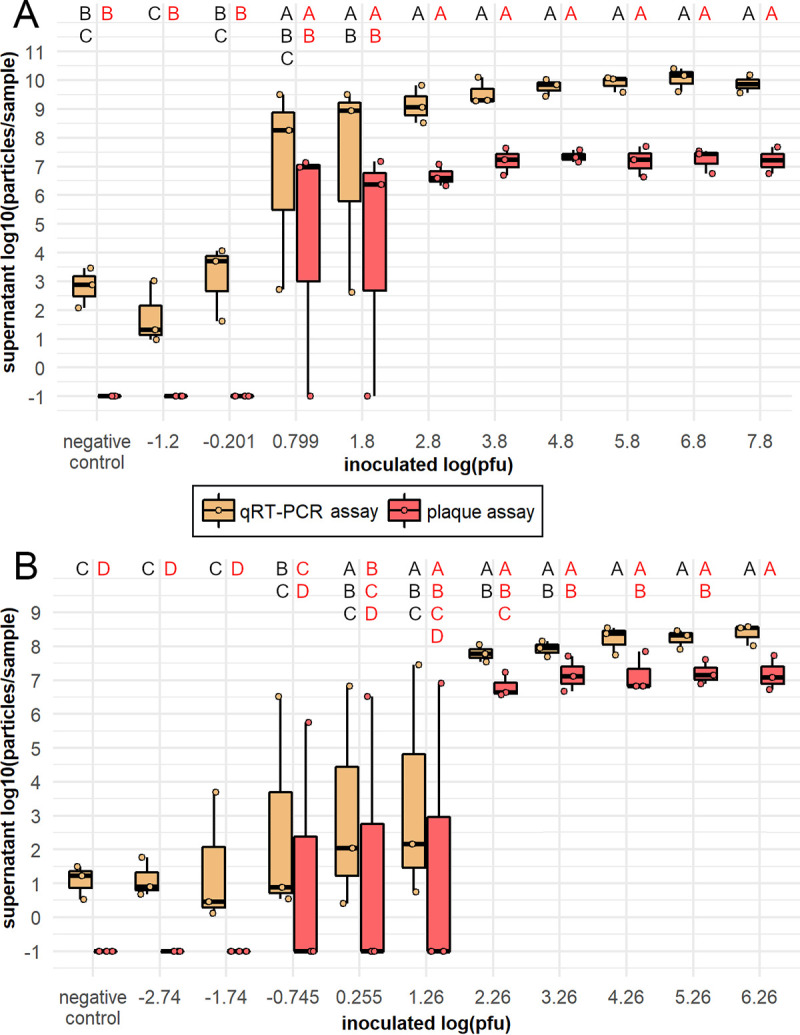
Infectious range of frozen ZIKV-Cambodia and ZIKV-Paraiba stocks *in vitro*. ZIKV Cambodia (A) and Paraiba (B) titers in C6/36 cell supernatants at 6 days post-inoculation with ZIKV stock at titers corresponding to qRT-PCR standard curve dilutions, or negative media control, displayed as log_10_ (particles/sample), measured by qRT-PCR (in log_10_ [p-e/sample]) and plaque assay (in log_10_ [PFU/sample + 0.1]). Titers are presented as ● symbols, and the boxplots indicate median titers and interquartile ranges. Results are from *N* = 2 to 3 and *N* = 3 independent biological replicates for ZIKV-Cambodia (A) and ZIKV-Paraiba (B), respectively. ZIKV titers were analyzed by ANOVA using the following model for ZIKV-Cambodia assayed by qRT-PCR and plaque assay and ZIKV-Paraiba assayed by qRT-PCR: Y_ij_ = μ + inoculum titer_j_; and using the following model for ZIKV-Paraiba assayed by plaque assay: Y_ijk_ = μ + inoculum titer_j_ + replicate_k_. Inoculum titer was a highly significant predictor of supernatant titer when assayed by qRT-PCR (ZIKV-Cambodia, *P* = 16.27E−06; ZIKV-Paraiba, *P* = 1.58E−05) or plaque assay (ZIKV-Cambodia, *P* = 8.94E−06; ZIKV-Paraiba, *P* = 7.21E−06), and this effect varied by replicate for ZIKV-Paraiba assayed by plaque assay (*P* = 0.029). The assumption of normality was violated in these analyses. Uppercase letters indicate significant differences between groups (*P* < 0.05) as determined by Tukey’s HSD *post hoc* analysis, with letters in black indicating comparisons between qRT-PCR-assayed titers and letters in red indicating comparisons between plaque assay titers.

For a mosquito to be competent for an arbovirus, transmission-relevant tissues must contain an infectious titer of virus. We therefore wanted to determine what titers of the ZIKV isolates used in this study constituted an infectious dose by using an informative but also accessible *in vitro* assay. We inoculated C6/36 mosquito cells, which lack a functioning RNA interference response (33, 41) and are highly susceptible to ZIKV infection, with a broad range ZIKV-Cambodia or Paraiba titers and identified which titers were infectious by assaying the cell culture supernatant at 6 days post-exposure (dpe) by qRT-PCR and plaque assay. We found that ZIKV titers in the supernatant varied significantly by inoculum titer when measured by both qRT-PCR (Analysis of Variance [ANOVA]: ZIKV-Cambodia, F = 9.96, *P* = 6.27E−06; ZIKV-Paraiba, F = 8.54, *P* = 1.58E−05) and plaque assay (ANOVA: ZIKV-Cambodia, F = 9.53, *P* = 8.94E−06; ZIKV-Paraiba, F = 9.79, *P* = 7.21E−06). Tukey’s honestly significant difference (HSD) *post hoc* analysis revealed that titers ≥2.80 log_10_ PFU for ZIKV-Cambodia (Fig. 1A) and ≥2.26 log_10_ PFU for ZIKV-Paraiba (Fig. 1B) always resulted in significantly higher ZIKV titers in cell supernatants compared with titers ≤−0.201 log_10_ PFU and ≤−1.74 log_10_ PFU, respectively. However, for ZIKV-Cambodia, ZIKV inoculum titers between −0.201 and 2.80 log_10_ PFU resulted in high supernatant ZIKV titers in 2 of 3 biological replicates (Fig. 1A). Likewise, for ZIKV-Paraiba, inoculums between −1.74 and 2.26 log_10_ PFU resulted in high titers of ZIKV in 1 of 3 replicates (Fig. 1B). We therefore concluded that, while samples with titers above these inoculum ranges were likely to contain infectious virus and therefore considered “positive” and samples below were unlikely to contain infectious virus and could be considered “negative,” the likely infectiousness of samples within this range could not be predicted by titer. Therefore, we categorized such samples as “indeterminate.” We found that the presence of infectious virus correlated directly with the presence of high titers of viral RNA and supported the application of the same positive/negative/indeterminate cut-offs (Fig. 1). We also observed signal in the supernatant of negative control samples inoculated with medium when assayed by qRT-PCR, suggesting potential background or low levels of contamination in the PCR assay. However, these samples contained no infectious virus; therefore, this did not impact the interpretation of the results. The efficiency, plate-to-plate variation, and correlation to RNA copy number derived from a full-length infectious viral clone (FLIC) for qRT-PCR standard curves for this study can be found in Fig. S1 in the supplemental material.

10.1128/mBio.01765-20.1 FIG S1 Standard curve mean *C_T_* values and copy number correlations across duplicate wells for all qRT-PCR plates included in this study. Mean *C_T_* values across duplicate wells for ZIKV-Cambodia (A), ZIKV-Paraiba (B), and FLIC standard curves (C) in log_10_ (PFU/reaction) for ZIKV-Cambodia and Paraiba, and log_10_ (copies/reaction) for FLIC. Linear fit lines correlating mean *C_T_* to log_10_ (PFU/reaction) or log_10_ (copies/reaction) are shown in blue with corresponding regression equations and *r*^2^ values. Calculated copy numbers of ZIKV-Cambodia (D) and ZIKV-Paraiba (E) standards expressed in terms of log_10_ (copies/reaction) as determined from linear fit line of FLIC standard curve. Linear fit lines calculating log_10_ (copies/reaction) from FLIC regression equation are shown in red with corresponding regression equation to convert mean *C_T_*s to log10 (copies/reaction) and *r*^2^ values. Mean *C_T_* values or log_10_ (copies/reaction) from duplicate wells are shown as ● symbols. The numbers above the points indicate the total number of times that step of the standard curve was assessed. Download FIG S1, TIF file, 0.2 MB. Copyright © 2020 MacLeod and Dimopoulos. 2020 MacLeod and Dimopoulos This content is distributed under the terms of the Creative Commons Attribution 4.0 International license.

### Multiple *C. quinquefasciatus* strains are not competent for ZIKV transmission.

**FIG 2:**
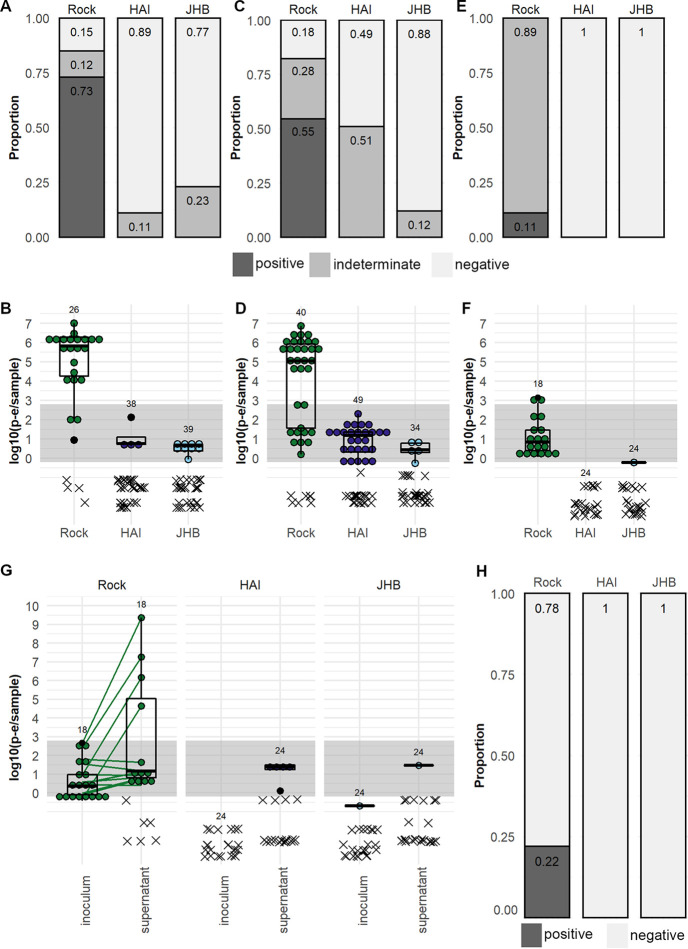
Vector competence of A. aegypti and *C. quinquefasciatus* for a pre-epidemic ZIKV isolate. ZIKV-Cambodia prevalence (A, C, and E) and titers (B, D, and F) in the midgut (A and B), salivary glands (C and D), and saliva (E and F) of A. aegypti Rock strain and *C. quinquefasciatus* HAI and JHB strains. (G) ZIKV-Cambodia titers in the inoculated saliva from A. aegypti Rock strain and *C. quinquefasciatus* HAI and JHB strains at 14 dpe and in the C6/36 cell supernatant at 6 days post-inoculation. (H) Prevalence of infectious (positive, dark gray) and non-infectious (negative, light gray) saliva samples by strain based on positive ZIKV outcomes in the C6/36 cell supernatant. Infectious virus prevalence in midguts, salivary glands, and saliva was classified as positive, indeterminate, or negative based on ZIKV-Cambodia limit of infectiousness *in vitro*. Titers are displayed as log_10_ (p-e/sample), with individual samples with detectable qRT-PCR product displayed as ● symbols and boxplots showing medians, interquartile ranges, and outliers by species; connecting lines indicate pre- and post-C6/36 RNA titers in paired samples. Samples without detectable qRT-PCR product are displayed as an x and were excluded from summary statistics. Samples falling within the gray bands are of indeterminate infection status; samples above the bands are considered positive, and samples below the bands are considered negative. Numbers above the data indicate total mosquitoes analyzed. Results are from *N* = 2 independent biological replicates for midguts, *N* = 2 to 4 independent biological replicates for salivary glands, and 1 replicate for saliva.

We determined the competence of *C. quinquefasciatus* JHB and HAI strains as well as A. aegypti Rock strain for ZIKV-Cambodia. ZIKV titers in log_10_ PFU equivalents (p-e)/sample in the midgut at 7 dpe were assessed by qRT-PCR, and the prevalence of infectious ZIKV was categorized based on the results of the C6/36 infection assay described above. We found that 73% of A. aegypti Rock strain midguts were positive at 7 dpe (Fig. 2A), and the average titer ± 95% confidence interval of ZIKV in Rock midgut samples with detectable PCR product was 5.12 ± 0.734 log_10_ (p-e/sample) (Fig. 2B). We found no positive midgut samples at 7 dpe in *C. quinquefasciatus* JHB or HAI strains. We found that 89% of JHB and 77% of HAI strain midguts were negative for ZIKV-Cambodia, and the remainder were of indeterminate infectious status (Fig. 2A); the average titers for both strains were ≤1 log_10_ (p-e/sample) (Fig. 2B). We concluded that these *C. quinquefasciatus* strains were highly resistant to ZIKV-Cambodia infection in the midgut.

We then investigated transmission by assessing ZIKV-Cambodia titers in the salivary glands and saliva of Rock, HAI, and JHB mosquitoes at 14 dpe. We found that 55% of salivary gland samples from A. aegypti strain Rock were positive (Fig. 2C), with an average titer of 4.22 ± 0.761 log_10_ (p-e/sample) (Fig. 2D). We found no positive salivary glands for either of the *C. quinquefasciatus* strains (Fig. 2C), but 12% of JHB and 51% of HAI salivary gland samples were of indeterminate infectious status. Average ZIKV titers in salivary gland samples from both *C. quinquefasciatus* strains were <1 log_10_ (p-e/sample) (Fig. 2D). The presence of indeterminate salivary gland samples suggested potential for dissemination of ZIKV particles in these strains of *C. quinquefasciatus*, and so we analyzed the ZIKV status of paired saliva samples collected from a subset of the mosquitoes. Despite the high prevalence of positive salivary glands, 89% of saliva samples from Rock mosquitoes were of indeterminate infection status (Fig. 2E); 11% were positive, and the average titer was 1.08 ± 0.470 log_10_ (p-e/sample) (Fig. 2F), indicating a bottleneck in ZIKV prevalence and titer between the salivary glands and saliva in A. aegypti Rock strain. We detected ZIKV in only 1 saliva sample from *C. quinquefasciatus* JHB strain, and the titer was <1 log_10_ p-e/sample; all other samples failed to amplify in the qRT-PCR (Fig. 2E and F). Summary statistics on the threshold cycle (*C_T_*) values obtained through qRT-PCR analysis of these samples can be found in Table S2.

10.1128/mBio.01765-20.10 TABLE S2 Summary statistics on *C_T_* values obtained through qRT-PCR analysis of midgut, salivary gland, and saliva samples from Rock, HAI, and JHB strain mosquitoes exposed to ZIKV, including mosquito strain, ZIKV isolate, exposure method, tissue type, days post-exposure (dpe), number of samples analyzed (*N*), mean *C_T_* value, and 95% confidence interval (ci). Download Table S2, DOCX file, 0.1 MB. Copyright © 2020 MacLeod and Dimopoulos. 2020 MacLeod and Dimopoulos This content is distributed under the terms of the Creative Commons Attribution 4.0 International license.

To confirm the presence or absence of infectious ZIKV in the saliva, we inoculated mosquito saliva samples on C6/36 cells and compared the ZIKV titer in the inoculum to that in the cell culture supernatant at 6 dpe and determined if the supernatants were positive for infectious ZIKV (≥2.80 log_10_ [p-e/sample]). We observed positive supernatants from 22% of Rock mosquito saliva samples; no saliva samples from either *C. quinquefasciatus* strain produced a positive result in the supernatant (Fig. 2G and H). We again observed some signal in the supernatants of negative controls (see Fig. S2), but it was consistent with the level described above (Fig. 1) which did not correspond to infectious ZIKV and therefore did not affect our conclusions about the infectiousness of saliva samples. We concluded that both strains of *C. quinquefasciatus* tested are not competent vectors for transmission of this virus.

10.1128/mBio.01765-20.2 FIG S2 Background signal in negative controls from C6/36 assay to detect infectious ZIKV *in vitro* for ZIKV vector competence assays. (A) Signals detected in medium-only controls in inoculum and in C6/36 cell supernatants after 6 days of incubation, expressed in log_10_ (p-e/sample) based on qRT-PCR standard curves. Individual samples with detectable qRT-PCR product are displayed as ● symbols, and the connecting line indicates pre- and post-C6/36 RNA titers in paired samples; samples without detectable qRT-PCR product are displayed as an x symbol. Numbers above the data indicate total *N* of samples analyzed. (B) Histograms showing counts per bin of mean *C_T_* values acquired from medium-only controls in inoculum and in C6/36 cell supernatants analyzed by qRT-PCR. Download FIG S2, TIF file, 0.1 MB. Copyright © 2020 MacLeod and Dimopoulos. 2020 MacLeod and Dimopoulos This content is distributed under the terms of the Creative Commons Attribution 4.0 International license.

**FIG 3:**
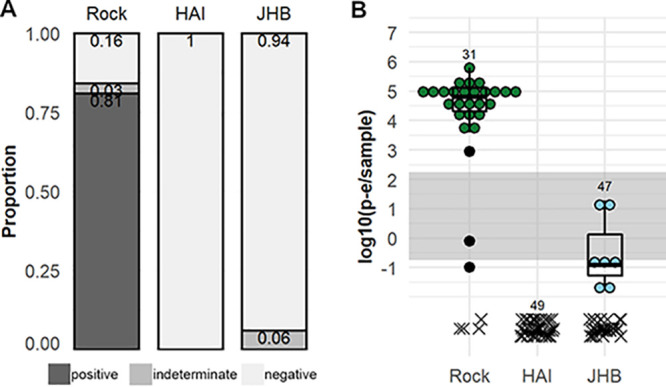
ZIKV isolate infection susceptibility of A. aegypti and *C. quinquefasciatus* for a post-epidemic ZIKV isolate. ZIKV-Paraiba prevalence (A) and titers (B) in the midguts of A. aegypti Rock strain and *C. quinquefasciatus* JHB and HAI strains. Prevalence is classified as positive, indeterminate, or negative based on ZIKV-Cambodia limit of infectiousness *in vitro*. Titers are displayed as log_10_ (p-e/sample), with individual samples with detectable qRT-PCR product displayed as ● symbols and boxplots showing medians, interquartile ranges, and outliers by species; samples without detectable qRT-PCR product are displayed as an x and are excluded from summary statistics. Samples falling within the gray band are of indeterminate infection status; samples above the band are considered positive, and samples below the band are considered negative. Numbers above the data indicate total mosquitoes analyzed. Results are from *N* = 2 independent biological replicates.

Since the *C. quinquefasciatus* HAI strain had previously been found competent for a post-epidemic ZIKV isolate, but was clearly not competent for ZIKV-Cambodia, a pre-epidemic strain (42), we wanted to address the possibility that these *C. quinquefasciatus* strains might be differentially competent for pre- and post-epidemic ZIKV isolates. We exposed mosquitoes to ZIKV-Paraiba, which is more similar in genome sequence to the ZIKV isolate previously found to infect the *C. quinquefasciatus* HAI strain than ZIKV-Cambodia (see Fig. S3) (28). Titers in the midgut at 7 dpe were assessed by qRT-PCR, and infectious ZIKV prevalence was categorized. We found that 81% of A. aegypti Rock strain samples were positive for ZIKV in the midgut at 7 dpe (Fig. 3A), and the average titer was 4.33 ± 0.604 log_10_ (p-e/sample) (Fig. 3B). We found no positive midguts for either the *C. quinquefasciatus* HAI or JHB strains; ZIKV was not detectable by qRT-PCR in midgut samples from HAI mosquitoes, and 6% of JHB midguts were of indeterminate status (Fig. 3A). The average titer for JHB midgut samples was <1 log_10_ (p-e/sample) (Fig. 3B). Summary statistics on *C_T_* values obtained by qRT-PCR analysis for ZIKV-Paraiba RNA in these samples can be found in Table S2. Based on these findings, we concluded that both *C. quinquefasciatus* strains tested are highly resistant to ZIKV-Paraiba infection in the midgut and unlikely to be competent vectors for this ZIKV isolate.

10.1128/mBio.01765-20.3 FIG S3 Phylogeny of ZIKV isolates used in studies of *Culex* species vector competence for ZIKV. Phylogenetic tree was built by the maximum likelihood method with GTR substitution model. Values at nodes represent bootstrap branch support out of 100 replications. ◀ ZIKV isolates used in this study; ◁ the ZIKV isolate previously tested in the HAI strain *C. quinquefasciatus*; ● other isolates of ZIKV found to infect *C. quinquefasciatus* in other studies. Download FIG S3, TIF file, 0.1 MB. Copyright © 2020 MacLeod and Dimopoulos. 2020 MacLeod and Dimopoulos This content is distributed under the terms of the Creative Commons Attribution 4.0 International license.

### ZIKV fails to replicate in the midgut of *C. quinquefasciatus*.

To identify the point at which ZIKV infection is arrested in *C. quinquefasciatus*, we compared the dynamics of ZIKV in the blood boluses and midguts of A. aegypti and *C. quinquefasciatus*. We assessed ZIKV-Cambodia levels in the blood boluses of A. aegypti Rock strain and *C. quinquefasciatus* JHB strain mosquitoes after blood feeding by plaque assay and qRT-PCR to determine if differential loss of infectious virus and/or viral RNA from the *C. quinquefasciatus* blood meal could explain resistance to ZIKV infection. However, we observed no significant difference in ZIKV dynamics between the blood boluses of Rock and JHB by plaque assay (ANOVA: F = 0.451, *P* = 0.508) (see Fig. S4, left); qRT-PCR analysis found higher titers of ZIKV RNA in blood boluses from JHB (ANOVA: F = 4.81, *P* = 0.038) (Fig. S4, right) which is likely explained by *C. quinquefasciatus* being larger than A. aegypti and taking larger blood meals (43, 44). These data suggested degradation in the blood bolus did not contribute to ZIKV infection resistance by *C. quinquefasciatus*.

10.1128/mBio.01765-20.4 FIG S4 Dynamics of pre-epidemic ZIKV in the blood boluses of A. aegypti and *C. quinquefasciatus*. ZIKV-Cambodia titers in the blood bolus of A. aegypti Rock strain (green) and *C. quinquefasciatus* JHB strain (blue) mosquitoes by plaque assay in log_10_ (average PFU/blood bolus + 0.1) (A) and qRT-PCR in log_10_ (average p-e/blood bolus) (B) from 0.5 to 48 hours post-exposure (hpe). Average titers per mosquito from pools of 25 mosquitoes per time point per replicate are presented as ● symbols, and boxplots indicate median titers and interquartile ranges. Samples falling within the gray band are of indeterminate infection status; samples above the band are considered positive, and samples below the band are considered negative. ZIKV titer limits for sample categories are adjusted to reflect pooled samples. Results are from *N* = 3 independent biological replicates. ZIKV titers in the blood bolus were analyzed by ANOVA using the following model: Y_ijk_ = μ + strain_j_ + hpe_k_. The effect of hpe was highly significant with both ZIKV detection methods (plaque assay, *P* = 2.65E−09; qRT-PCR, *P* = 4.13E−08); ZIKV titers differed by strain when measured by qRT-PCR (*P* = 0.38) but not plaque assay (*P* = 0.552). The assumption of normality was violated. Uppercase letters indicate significant difference between groups (*P* < 0.05) as determined by Tukey’s HSD *post hoc* analysis. Download FIG S4, TIF file, 0.1 MB. Copyright © 2020 MacLeod and Dimopoulos. 2020 MacLeod and Dimopoulos This content is distributed under the terms of the Creative Commons Attribution 4.0 International license.

**FIG 4:**
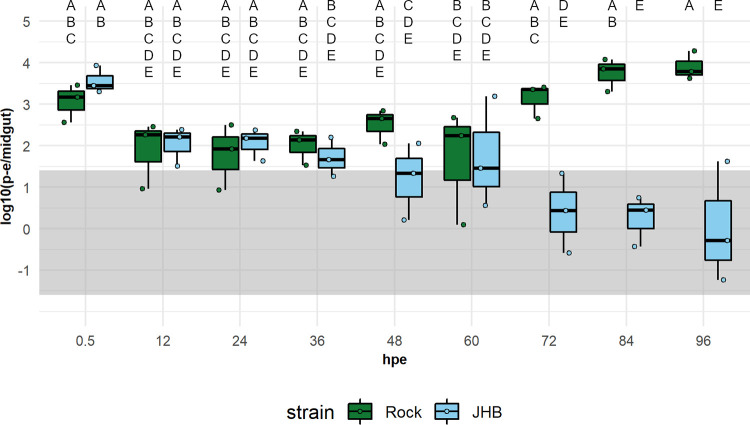
Dynamics of pre-epidemic ZIKV in the midguts of A. aegypti and *C. quinquefasciatus*. ZIKV-Cambodia titers in the midguts of A. aegypti Rock strain (green) and *C. quinquefasciatus* JHB strain (blue) mosquitoes by qRT-PCR in log_10_ (mean p-e/midgut) at 12-hour intervals post-exposure. Average titers per mosquito from pools of 25 mosquitoes per time point per replicate are presented as ● symbols, and boxplots indicate median titers and interquartile ranges. Samples falling within the gray band are of indeterminate infectious status; samples above the band are considered positive, and samples below the band are considered negative. ZIKV titer limits for sample categories are adjusted to reflect pooled samples. Results are from *N* = 3 independent biological replicates. ZIKV titers in the midgut were analyzed by ANOVA using the following model: Y_ijklm_ = μ + strains_j_ + hpe_k_ + replicate_l_ + species:hpe_m_. In the midgut analysis, there was a significant interaction between strain and hpe (*P* = 0.006) and a significant effect of replicate (*P* = 0.012). The assumption of normality was violated. Uppercase letters indicate significant difference between groups (*P* < 0.05) as determined by Tukey’s HSD *post hoc* analysis.

We then assessed viral dynamics over time in the midgut of *C. quinquefasciatus* to determine the point at which ZIKV infection is arrested. We quantified ZIKV-Cambodia levels in the midguts of *C. quinquefasciatus* JHB and A. aegypti Rock strain mosquitoes every 12 hours for 96 hours post-exposure (hpe) by qRT-PCR. We found that ZIKV dynamics varied significantly between JHB and Rock mosquitoes (ANOVA: F = 9.47, *P* = 9.04E−07). Tukey’s HSD *post hoc* analysis confirmed that ZIKV titers were similar in Rock and JHB midguts from 0.5 to 60 hpe, exhibiting a drop in titer from 0.5 to 12 hpe (Fig. 4). However, after 60 hpe, ZIKV levels in Rock rose, exhibiting the post-eclipse period pattern of RNA replication, whereas ZIKV titers in JHB declined after 60 hpe to below the cut-off for positive samples by 72 hpe (Fig. 4). We therefore concluded that ZIKV failed to replicate in the midgut tissue of *C. quinquefasciatus* and that this at least in part explained the barrier to infection in this mosquito species.

### There are multiple tissue barriers to ZIKV transmission in *C. quinquefasciatus* mosquitoes.

**FIG 5:**
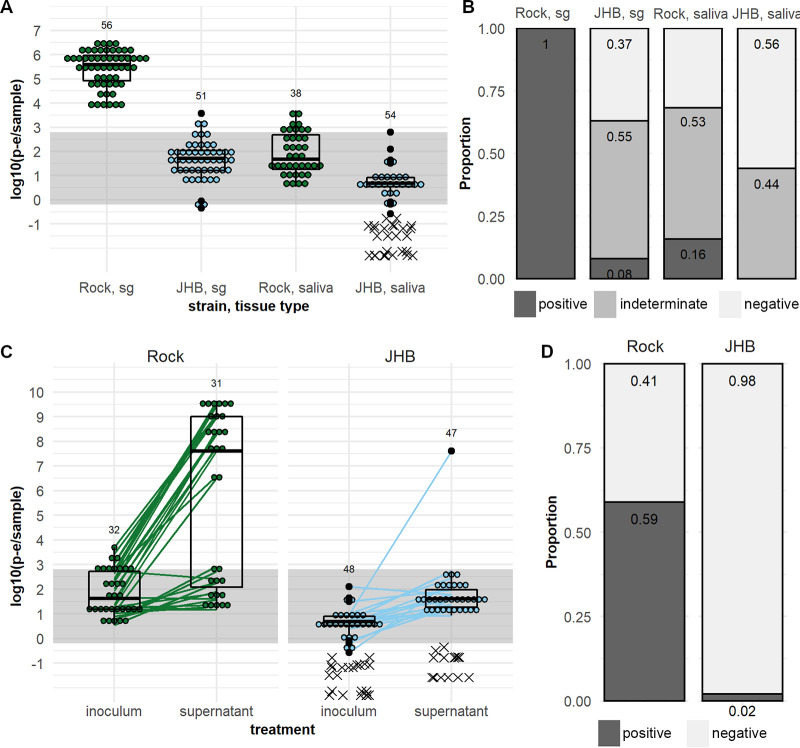
Salivary gland and saliva transmission of pre-epidemic ZIKV by A. aegypti and *C. quinquefasciatus* after exposure by intrathoracic injection. ZIKV-Cambodia prevalence (A) and titers (B) in the salivary glands, and saliva of A. aegypti Rock strain and *C. quinquefasciatus* JHB strain at 7 dpi. (C) ZIKV-Cambodia titers in the inoculated saliva from A. aegypti Rock strain and *C. quinquefasciatus* HAI and JHB strains at 14 dpi and in the C6/36 cell supernatant at 6 days post-inoculation. (D) Prevalence of infectious (positive, dark gray) and non-infectious (negative, light gray) saliva samples by strain based on positive ZIKV outcomes in the C6/36 cell supernatant. Titers are displayed as log_10_ (p-e/sample), with individual samples with detectable qRT-PCR product displayed as ● symbols and boxplots showing medians, interquartile ranges, and outliers by species; connecting lines indicate pre- and post-C6/36 RNA titers in paired samples. Samples without detectable qRT-PCR product are displayed as an x and were excluded from summary statistics. Samples falling within the gray bands are of indeterminate infection status; samples above the band are considered positive, and samples below the band are considered negative. Numbers above the data indicate total mosquitoes analyzed. Results are from *N* = 2 independent biological replicates.

To determine if bypassing the midgut would allow ZIKV transmission by *C. quinquefasciatus*, we exposed A. aegypti Rock strain and *C. quinquefasciatus* JHB strain mosquitoes to ZIKV by injecting the virus intrathoracically. We measured ZIKV titers in the salivary glands and saliva of injected mosquitoes at 7 days post-injection (dpi) by qRT-PCR. We found that 100% of salivary glands in injected Rock mosquitoes were positive for infectious ZIKV based on titer, whereas only 8% of JHB salivary glands were positive (Fig. 5A). The average ZIKV titer in JHB salivary glands of 1.67 ± 0.207 log_10_ (p-e/sample) was lower than in Rock salivary glands at 5.42 ± 0.199 log_10_ (p-e/sample) (Fig. 5B). Prevalence of positive samples was lower in Rock saliva than salivary glands; 16% of Rock saliva samples were positive (Fig. 5A). The average ZIKV titer in the Rock saliva was 1.90 ± 0.285 log_10_ (p-e/sample) (Fig. 5B). In JHB, no saliva samples were positive for infectious ZIKV by titer; 44% were negative, and 56% were of indeterminate status (Fig. 5A), with an average ZIKV titer <1 log_10_ (p-e/sample) (Fig. 5B). Summary statistics on the *C_T_* values obtained by qRT-PCR analysis for ZIKV-injected mosquito samples can be found in Table S2.

While the low ZIKV titer in the saliva of injected JHB compared to that in Rock mosquitoes did not suggest the presence of infectious virus, we wanted to confirm the presence or absence of virions in the saliva of injected mosquitoes. We therefore inoculated saliva from Rock and JHB samples on C6/36 cells and detected ZIKV in the supernatants at 6 dpe by qRT-PCR. For the A. aegypti Rock strain, 59% of inoculated saliva samples produced positive supernatants at 6 dpe (Fig. 5C and D). For the JHB strain *C. quinquefasciatus*, only 1 saliva sample produced a ZIKV-positive supernatant (Fig. 5C and D). Similar to that in previous assays, the signal in uninfected controls was inconsistent with the presence of infectious ZIKV (see Fig. S5). In summary, we concluded that ZIKV RNA was detectable in salivary gland and saliva samples of the injected *C. quinquefasciatus* JHB strain, but this was rarely followed by dissemination of infectious virus in the saliva. We therefore concluded that ZIKV transmission by *C. quinquefasciatus* mosquitoes is impeded at the level of the midgut and salivary glands.

10.1128/mBio.01765-20.5 FIG S5 Background signal in negative controls from C6/36 assay to detect infectious ZIKV *in vitro* for ZIKV injection assays. (A) Signals detected in medium-only controls in inoculum and in C6/36 cell supernatants after 6 days of incubation, expressed in log_10_ (p-e/sample) based on qRT-PCR standard curves. Individual samples with detectable qRT-PCR product are displayed as ● symbols, and the connecting line indicats pre- and post-C6/36 RNA titers in paired samples; samples without detectable qRT-PCR product are displayed as an x symbol. Numbers above data indicate total *N* of samples analyzed. (B) Histograms showing counts per bin of mean *C_T_* values acquired for medium-only controls in inoculum and in C6/36 cell supernatants analyzed by qRT-PCR. Download FIG S5, TIF file, 0.1 MB. Copyright © 2020 MacLeod and Dimopoulos. 2020 MacLeod and Dimopoulos This content is distributed under the terms of the Creative Commons Attribution 4.0 International license.

### ZIKV may enter non-susceptible *Culex* cell lines *in vitro*.

Given the prolonged persistence of ZIKV in the midguts of *C. quinquefasciatus* without evidence of viral replication, we hypothesized that ZIKV may enter *Culex* cells that do not support replication and be blocked downstream of cell entry. We attempted to identify virus particles in Culex tarsalis Chao Ball (ChaoB) cells, which do not support ZIKV infection (45), as well as A. aegypti Aag2 cells, and in A. aegypti Rock strain and *C. quinquefasciatus* JHB strain midguts by transmission electron microscopy (TEM), which has been used to identify ZIKV particles in mosquitoes in other studies (29). However, in the ZIKV-exposed (see Fig. S6) and unexposed (see Fig. S7) samples from both *Aedes* and *Culex* cells and tissues, we observed objects resembling flaviviruses. Given the potential for mosquito cell lines and tissues to be persistently infected with insect-specific flaviviruses (ISFVs) (46–51), we found TEM data inconclusive regarding ZIKV localization in mosquito samples.

10.1128/mBio.01765-20.6 FIG S6 Particles resembling flaviviruses in cells and tissues from A. aegypti and *C. quinquefasciatus*. TEM images of cell lines (A to F) and midgut tissues (G to J) of *Aedes* and *Culex* mosquitoes after exposure to ZIKV-Cambodia. (A to F) Aag2 and ChaoB cell lines were exposed to ZIKV-Cambodia at an MOI of 100 and fixed at 15 minutes post-exposure (mpe). (G to J) Rock and JHB mosquitoes were exposed to ZIKV at 2.1E09 PFU/ml via blood feeding for 30 minutes, and midguts with blood boluses were dissected at 30 mpe. In these midgut samples, prominent structures of blood bolus (bb), basal lamina (bl), vesicles (vs), endoplasmic reticulum (er), and mitochondria (mt) are labeled in white, and vertical diameters (in nanometers) of particles resembling flavivirus are labeled in red. Bars, 100 nm for cell images and 200 nm for midgut images. Download FIG S6, TIF file, 0.5 MB. Copyright © 2020 MacLeod and Dimopoulos. 2020 MacLeod and Dimopoulos This content is distributed under the terms of the Creative Commons Attribution 4.0 International license.

10.1128/mBio.01765-20.7 FIG S7 TEM images of uninfected controls from mosquito cells and tissues. Images of cell lines (A to F) and midgut tissues (G to J) of uninfected *Aedes* and *Culex* mosquitoes. (A to F) Aag2 and ChaoB cell lines were treated with medium containing no virus and fixed at 15 mpe. (G to J) Rock and JHB mosquitoes were fed on uninfected blood meals containing C6/36 cell culture medium for 30 minutes, and midguts with blood boluses were dissected at 30 mpe. In these midgut samples, prominent structures of blood bolus (bb), red blood cells (rbc), basal lamina (bl), vesicles (vs), endoplasmic reticulum (er), cell junctions (cj), and mitochondria (mt) are labeled in white, and vertical diameters (in nanometers) of cellular structures that resembled flavivirus particles are labeled in white. Bars, 100 nm for cell images and 200 nm for midgut images. Download FIG S7, TIF file, 0.5 MB. Copyright © 2020 MacLeod and Dimopoulos. 2020 MacLeod and Dimopoulos This content is distributed under the terms of the Creative Commons Attribution 4.0 International license.

**FIG 6:**
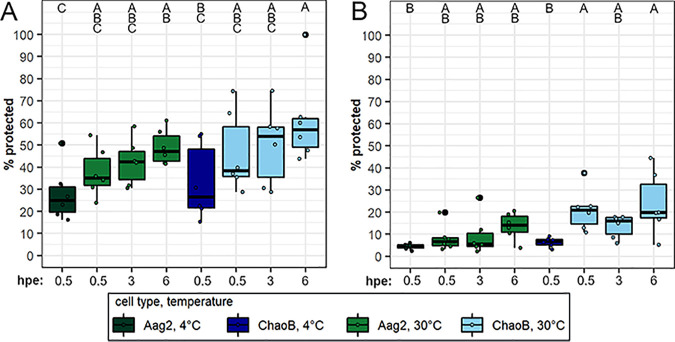
Internalization dynamics of pre-epidemic ZIKV in *Aedes* and *Culex* cells *in vitro.* ZIKV-Cambodia protection from proteinase K (A) and trypsin (B) protease treatment in A. aegypti Aag2 and *C. tarsalis* ChaoB cell lines following incubation at internalization-inhibiting and -permissive temperatures at 0.5, 3, and 6 hpe. Percentage virus protected is the ratio of ZIKV remaining in protease treatment wells compared to that in paired untreated control wells, with 3 pairs per replicate, and is presented as a ● symbol with boxplots indicating median titers and interquartile ranges. Conditions incubated at 4°C to inhibit internalization are shown in dark green and dark blue for Aag2 and ChaoB cells, respectively. Conditions incubated at 30°C to allow internalization are shown in light green and light blue for Aag2 and ChaoB cells, respectively. Results are from *N* = 2 independent biological replicates. Percent protected ZIKV in proteinase K treatments was analyzed following log_10_ transformation by ANOVA using the following model: Y_ijkl_ = μ + cell type_j_ + temperature_k_ + hpe_l_. The effect of temperature was highly significant (*P* = 7.07E−05). There was a moderate but non-significant effect of cell type (*P* = 0.107) and hpe (*P* = 0.112). Percent protected ZIKV in trypsin treatments was analyzed following log_10_ transformation by ANOVA using the following model: Y_ijkl_ = μ + cell type_j_ + temperature_k_ + hpe_l_. There was a highly significant effect of both temperature (*P* = 9.96E-05) and cell type (*P* = 6.5E-04). There was a moderate but non-significant effect of hpe (*P* = 0.114). Uppercase letters indicate significant differences between groups (*P* < 0.05) as determined by Tukey’s HSD *post hoc* analysis.

We therefore investigated ZIKV entry into *Aedes* and *Culex* cell lines *in vitro*. We used proteinase K or trypsin treatment to remove extracellular virus and assess internalization of ZIKV in Aag2 and ChaoB cells at internalization-inhibiting and permissive temperatures over time. In proteinase K assays, we found that significantly more virus was protected from treatment at internalization-permissive temperatures than at internalization-inhibiting temperatures (ANOVA: F = 19.3, *P* = 7.07E−15) and that there was a trend toward increasing protection from treatment with time that was not statistically significant (ANOVA: F = 2.31, *P* = 0.112). Tukey’s HSD *post hoc* analysis revealed that there was a significant increase in percent ZIKV protected in both Aag2 and ChaoB cells (Fig. 6A), which supported the hypothesis that ZIKV is internalized, therefore being protected from protease treatment, in *Culex* cells. We observed a similar pattern in the trypsin assay (Fig. 6B). Significantly more virus was protected from trypsin treatment at internalization-permissive temperatures than at internalization-inhibiting temperatures (ANOVA: F = 18.4, *P* = 9.96E−05), and there was a non-significant trend toward increasing protection from treatment over time (ANOVA: F = 2.29, *P* = 0.114). Pairwise comparisons via Tukey’s HSD *post hoc* analysis revealed there was a significant increase in the percentage of ZIKV protected from trypsin in ChaoB cells at internalization-permissive temperatures (Fig. 6B). In Aag2 cells, there was an increase in the percentage of ZIKV protected (Fig. 6B), though this trend was not significant. A comparison of total RNA levels in proteinase K- and trypsin-treated cells can be found in Fig. S8. Overall, these results support the hypothesis of ZIKV internalization by *Culex* cells.

10.1128/mBio.01765-20.8 FIG S8 RNA concentrations in protease-treated and control cells from *in vitro* ZIKV internalization assays. Total RNA (in ng/μl) in proteinase K-treated (A) and trypsin proteinase-treated (B) A. aegypti Aag2 and *C. tarsalis* ChaoB cell lines. RNA values are presented as ● symbols, and boxplots indicate median titers and interquartile ranges, with 3 replicate wells per condition per independent biological replicate. Results are from *N* = 2 independent biological replicates. RNA concentration in proteinase K treatments was analyzed following log_10_-transformation of RNA concentration by ANOVA using the following model: Y_ijk_ = μ + cell type_j_ + treatment_k_. The effect of cell type (*P* = 1.42E−12) and treatment (*P* < 2E−16) were highly significant. RNA concentration in trypsin treatments was analyzed following log_10_-transformation of RNA concentration by ANOVA using the following model: Y_ijkl_ = μ + cell type_j_ + treatment_k_ + cell type:treatment_l_. There was a significant interaction between the effects of cell type and treatment (*P* = 6.62E−13). Uppercase letters indicate significant difference between groups (*P* < 0.05) as determined by Tukey’s HSD *post hoc* analysis. Download FIG S8, TIF file, 0.1 MB. Copyright © 2020 MacLeod and Dimopoulos. 2020 MacLeod and Dimopoulos This content is distributed under the terms of the Creative Commons Attribution 4.0 International license.

## DISCUSSION

Using robust methodology, we determined that neither the *C. quinquefasciatus* JHB nor HAI strain is susceptible to infection by pre- or post-epidemic ZIKV isolates, and we found that both of these strains fail to transmit infectious ZIKV in the saliva. We therefore conclude they are not competent for ZIKV transmission. While we found a clear barrier to ZIKV infection in the midgut and to the dissemination of infectious virions from the salivary glands of *C. quinquefasciatus*, we were able to detect ZIKV RNA in transmission-relevant tissues in these mosquitoes for prolonged periods post-exposure. Based on results from *in vitro* assays, we hypothesize that ZIKV is internalized by *Culex* and *Aedes* cells similarly and that infection is blocked downstream of cell entry.

The majority of studies on the potential for ZIKV transmission by *C. quinquefasciatus* and other *Culex* mosquito species have concluded that these mosquitoes are not competent vectors of ZIKV (see Table S1 in the supplemental material). However, in our study and in others, low levels of ZIKV RNA (22, 23, 52–56) and even infectious virus (45, 57, 58) can be detected in *Culex* mosquitoes despite a lack of ZIKV transmission in the saliva. Given the failure of ZIKV to replicate in the midgut (Fig. 4), we concluded that a robust midgut infection barrier in part prevents the transmission of ZIKV by *C. quinquefasciatus*. However, ZIKV RNA levels are similar in both susceptible A. aegypti and resistant *C. quinquefasciatus* for many hours post-exposure. Other studies investigating *C. quinquefasciatus* competence for ZIKV observed low levels of ZIKV RNA in these mosquitoes many days post-blood feeding (45, 53–56, 58). It has been suggested that a lack of virus infection in the midguts of refractory mosquito species is due to a lack of appropriate viral receptors on the apical epithelial membrane (13, 59). Additionally, it has been observed that arboviruses can bind to the epithelial membranes of both susceptible and refractory mosquitoes but that binding is nonspecific in refractory mosquitoes (13). Persistent detection of ZIKV in the midgut suggests that this virus may be able to bind to cells of *C. quinquefasciatus*, but how this impacts infection barriers remains unclear.

We also determined that ZIKV transmission by *C. quinquefasciatus* is blocked at the salivary gland level. When we injected mosquitoes with ZIKV to bypass the midgut infection stage, *C. quinquefasciatus* salivary gland and saliva titers were low compared to those of A. aegypti, and saliva from *C. quinquefasciatus* was rarely infectious. However, our data suggest that ZIKV particles in the hemocoel may infrequently disseminate in saliva of injected *C. quinquefasciatus*. Our findings agree with other studies that found no evidence of ZIKV transmission by *Culex* species after intrathoracic injection of virus (45, 58). However, a study of injected *C. restuans* and *C. tarsalis* found that while no ZIKV RNA was detected in saliva from *C. tarsalis*, some *C. restuans* saliva samples were positive for ZIKV RNA; virus infectiousness was not assessed (60). ZIKV infection and transmission by *C. quinquefasciatus* is clearly obstructed at the midgut and salivary glands, indicating a systemic barrier to ZIKV infection and dissemination in *C. quinquefasciatus*, but it is not clear if this is the case in other *Culex* species.

qRT-PCR is a rapid and sensitive way to determine if ZIKV RNA can be found in different mosquito species and tissues. However, PCR-based detection of viral nucleic acid does not necessarily indicate the presence of infectious virus, and detection of arbovirus nucleic acids within a mosquito does not prove vector competence (38–40, 61). For a mosquito to serve as a competent vector for an arbovirus, the virus must replicate in various mosquito tissues (8, 62, 63); therefore, detection of infectious virions is critical to declaring a mosquito “competent.” Correlating the virus titers in mosquito tissue samples measured by qRT-PCR with what is was expected to be an infectious dose of ZIKV *in vitro* led us to classify samples in 3 infectiousness categories: positive, negative, and indeterminate. Based on this assessment, while *C. quinquefasciatus* tissue samples sometimes contained ZIKV RNA, the low viral titer of these samples (within the negative and indeterminate ranges) necessitated additional investigation of the mosquito saliva which conclusively demonstrated that these mosquitoes failed to transmit virions. These findings indicate that not all mosquito samples in which ZIKV RNA can be detected should be considered “positive” in the context of vector competence and highlight the value of including assays for infectious virus when assessing mosquito competence for arboviruses.

*In vitro* methods such as C6/36 cell and plaque assays to detect infectious virus are simple and accessible ways to assess vector competence. However, the amount of virus required to establish infection in these models may be less relevant than *in vivo* assays using vertebrate hosts. In natural infections, interactions between arboviruses, mosquito saliva, and the animal host impact viral infection and pathogenicity in animal models (64). While the minimum inoculum of ZIKV required to establish infection in a mammalian host has not been specifically established and likely varies by a multitude of factors such as viral strain and host species, studies in primates found that A. aegypti with 1.5 to 3.2 log_10_ PFU ZIKV in the saliva infected 100% of rhesus macaques after multiple probing attempts by single mosquitoes (65). This range is similar to the dose required to produce infection in C6/36 cells in the majority of biological replicates (Fig. 1). Since the use of animal models is not always feasible, the need for alternative methods of demonstrating viral transmission by mosquitoes has long been recognized (66). The use of *in vitro* assays to establish infectious doses of arboviruses improves interpretation of qRT-PCR-based studies of vector competence, but further studies on the impacts of mosquito saliva and host on minimum infectious doses of ZIKV and other arboviruses are needed.

Contrary to the majority of the literature, 4 studies concluded that different strains of *C. quinquefasciatus* are competent vectors of various ZIKV isolates (28–31). It has been suggested that strain specificity in *C. quinquefasciatus* competence for diverse ZIKV isolates could explain this discrepancy (25, 31). Studies on *C. quinquefasciatus* from Recife, Brazil, identified a high titer of a ZIKV isolate collected in Brazil (PE243, GenBank KX197192.1) in the midguts of these mosquitoes, although no increase in ZIKV titer was observed post-blood feeding (29), suggesting an absence of viral replication. The *C. quinquefasciatus* HAI strain collected from Hainan Province, China, was previously found to be competent for a ZIKV isolate collected from a patient traveling from American Samoa (SZ01, GenBank KU866423), and ZIKV RNA was detected in the brains of neonate mice after being fed on by HAI strain mosquitoes; infectious virus from mouse samples was not reported (28). We found these mosquitoes to be highly resistant to infection with both pre- and post-epidemic ZIKV isolates and detected no infectious ZIKV-Cambodia in their saliva. The opportunity to further test the susceptibility of this *C. quinquefasciatus* strain was incredibly valuable for addressing the hypothesis of intraspecific variability in ZIKV competence; however, we were unable to retest the same ZIKV isolate used in the original study (28). Therefore, it is possible that this mosquito strain is simply not susceptible to the ZIKV isolates we used. *C. quinquefasciatus* collected in Vero Beach, FL, were found to be susceptible to infection with a ZIKV isolate from Puerto Rico (PRVABC59, GenBank KU501215.1). Body titers were often low, but saliva collected on filter cards from pooled mosquitoes was positive by plaque assay (31). Given the stochasticity of viral infection, which we observed in our assays on infectious ZIKV titers (Fig. 1) (67), a very small amount of ZIKV may be infectious in some cases, which could explain the positive saliva from ZIKV-exposed Florida *C. quinquefasciatus*. Notably, saliva titers in these mosquitoes were highly variable between replicates (31). However, the large number of studies demonstrating *C. quinquefasciatus* to be not competent for PRVABC59 and other ZIKV isolates (Table S1) weakens the hypothesis of intraspecific variation in *C. quinquefasciatus* competence for different ZIKV isolates. To fully address this biological explanation for the discrepancy in findings on *C. quinquefasciatus* competence for ZIKV, increased sharing of mosquito strains and virus isolates would be necessary between members of the vector biology community.

The process by which arboviruses are blocked in mosquitoes at the cellular and molecular levels is poorly understood. Most mechanistic investigations into these barriers have focused on the identification of receptors required for arbovirus binding to susceptible cell membranes (10, 68–72). However, little emphasis has been placed on characterizing how host-virus interactions differ in refractory mosquito species. Given the persistent detection of ZIKV in *C. quinquefasciatus* tissues despite this species’ resistance to infection in our study and others (22, 23, 52–56), we hypothesized that ZIKV may be blocked downstream of cell entry in *Culex* mosquitoes but found TEM to be insufficient to address this question. Guedes et al. used TEM to identify ZIKV particles in the salivary glands of *C. quinquefasciatus* and also observed cytopathic disruptions deemed consistent with viral infection (29); however, the high potential for mosquito cells and tissues to be persistently infected with ISFVs, which would be impossible to distinguish from ZIKV by TEM, hampers the use of TEM for the virus-specific identification of flavivirus particles in mosquito cells (46–51). We therefore assessed virus internalization in cells by *in vitro* methods to test our hypothesis that ZIKV infection in *Culex* cells was blocked downstream of cell entry.

Our finding that the proportion of ZIKV protected from protease treatment increases with incubation at internalization-permissive temperatures in both *Aedes* and *Culex* cell lines supports the hypothesis of cell entry. The possibility that ZIKV is internalized in non-susceptible *Culex* cells has important implications for the nature of the infection barrier in *C. quinquefasciatus* and other *Culex* species. Few studies have attempted to dissect internalization and infection in pathogenic arbovirus barriers, but work in ISFVs and other host-pathogen systems suggests viruses may be restricted in non-susceptible cell types after attachment and entry (73–77). These studies support the hypothesis that factors downstream of cell entry, such as host-factor availability or antiviral response, play a critical role in determining the susceptibility of different arthropod species to viral infection and highlight the need for further investigation of this hypothesis *in vivo*, not only in *C. quinquefasciatus* resistance to ZIKV infection but also in determining the vector range of other arboviruses.

The potential for other vector species, such as *Culex* mosquitoes, to transmit ZIKV was originally proposed as a possible explanation for the severity of the ZIKV epidemic in the Americas in 2015 to 2016 (24, 25). The preponderance of data suggests no major role for *C. quinquefasciatus* in ZIKV transmission. While the hypothesis remains for intraspecific variability in *C. quinquefasciatus* ZIKV competence, differences in methodological approaches to virus detection likely drive the discrepancy in the literature on this topic. This study highlights the benefit of the exchange of material between investigators in this field in addressing intraspecific variability in vector competence; however, increased collaboration between research groups is necessary to truly address these questions. Based on the current evidence of this study and others, transmission by *C. quinquefasciatus* is highly unlikely to explain the severity or pace of the American ZIKV epidemic. We have identified the barriers to ZIKV infection in *C. quinquefasciatus* mosquitoes and offered a starting point for the renewed investigation of mechanisms by which arboviral infection is disrupted in refractory mosquito species.

## MATERIALS AND METHODS

### Ethics statement.

This study was carried out in accordance with the recommendations in the Guide for the Care and Use of Laboratory Animals of the National Institutes of Health, the Animal Care and Use Committee of the Johns Hopkins University, and the institutional ethics committee (permit MO18H82). Mice were only used for mosquito rearing. Commercial, anonymous human blood was used for virus infections in mosquitoes; therefore, informed consent was not applicable.

### Cell culture.

The *A. albopictus* cell line C6/36 was cultured in minimal essential medium (MEM; Gibco, catalog no. 11095-080) that was supplemented with 10% fetal bovine serum (FBS; Sigma, catalog no. F4135), 2 mM l-glutamine (Gibco, catalog no. 25030-081), 1% penicillin (10,000 units/ml)-streptomycin (10,000 μg/ml) (P/S; Gibco, catalog no. 15140-122), and 1% nonessential amino acids (Gibco, catalog no. 11140-050) and incubated at 32°C with 5% CO_2_. The A. aegypti cell line Aag2 and *C. tarsalis* Chao Ball (ChaoB) cell line were cultured in Schneider’s *Drosophila* medium (Gibco, catalog no. 21720-024) supplemented with 10% FBS and 1% P/S. The Aag2 line was incubated at 30°C with no CO_2_, and the ChaoB line was incubated at 32°C with 5% CO_2_. Baby hamster kidney (BHK-21) and Vero cells were maintained in Dulbecco’s modified Eagle’s medium (DMEM; Gibco, catalog no. 11995065) supplemented with 10% FBS, 1% l-glutamine, 1% P/S, and 5 μg/ml Plasmocin (InvivoGen, catalog no. ant-mpp) and incubated at 37°C with 5% CO_2_.

### Mosquito rearing.

A. aegypti Rockefeller strain (Rock) and *C. quinquefasciatus* Johannesburg strain (JHB, NR-43025; BEI Resources, Johannesburg, South Africa) colonies were fed on anaesthetized Swiss-Webster female mice for 30 minutes to 1 hour for egg production; *C. quinquefasciatus* HAI strain (Hainan, China) (28) was fed overnight on a 1:1 mixture of washed human red blood cells and serum from anonymous donors. Larvae for all mosquito strains were provided larval food (liver powder, tropical fish flake food, and rabbit food pellets mixed at a 2:1:1 ratio) *ad libitum* until pupation. Post-eclosion, both A. aegypti and *C. quinquefasciatus* mosquito colonies were maintained on 10% sucrose solution *ad libitum* at 27°C and 80% relative humidity, with a 14:10-hour light:dark cycle.

### Virus propagation and titration.

Virus propagation was carried out under biosafety level 2 laboratory conditions. Both pre-epidemic ZIKV strains Cambodia (ZIKV-Cambodia) passage 2 or 4 (FSS13025, GenBank JN860885) (42) and post-epidemic Paraiba (ZIKV-Paraiba) (78) passage 2 (Paraiba_01, GenBank KX280026) were propagated in C6/36 cells for 6 days, at which time the viruses were harvested at passage 3 or 5 and stored at −80°C in 1% SPG buffer (2.18 M sucrose, 38 mM KH_2_PO_4_, 72 mM K_2_HPO_4_, 60 mM l-glutamic acid). Virus titration was performed via plaque assay as previously described (79); in brief, frozen virus stocks were thawed at room temperature, and serial dilutions were inoculated on BHK-21 monolayers. Cells were fixed in methanol-acetone and 1% crystal violet after 4 days, and the numbers of plaques were counted to determine viral titer.

### Viral infection assays.

All virus infection assays and collection of potentially infected tissues were carried out under arthropod containment level 3 conditions. For oral infection assays, mosquitoes were starved 6 to 9 hours prior to infection. Frozen ZIKV stocks were thawed at room temperature and diluted 1:1 with washed human red blood cells (Interstate Blood Bank, Inc.); infectious blood meals were supplemented with 10% (vol/vol) human serum (Interstate Blood Bank, Inc.) and 10 mM ATP. According to virus titration and subsequent dilution, ZIKV-Cambodia was fed to mosquitoes at between 8.5E06 and 2.1E09 PFU/ml, and ZIKV-Paraiba was fed at 6.0E07 PFU/ml. Water-jacketed glass membrane feeders were used to hold blood meals and were maintained at 37°C. Mosquitoes aged 5 to 7 days post-eclosion were allowed to feed for 30 minutes, after which mosquitoes were cold anaesthetized, and unfed females were removed. After exposure to ZIKV, mosquitoes were housed in reach-in incubators at 27°C with 80% relative humidity until dissections.

For injection infection assays, Rock and JHB strain mosquitoes, aged 5 to 7 days post-eclosion, were injected intrathoracically with 0.69 nl of thawed viral stock, amounting to 2.9E05 PFU of ZIKV-Cambodia passage 3. Injections were performed using a Nanoject II Auto-Nanoliter injector (Drummond Scientific). Mosquitoes were allowed to recover under increased local humidity until 3 days post-injection (dpi), and housed under standard humidity conditions until sample collection. At 7 dpi, saliva was collected from individual mosquitoes as described below. Salivary glands of injected mosquitoes were dissected individually after saliva collection as described below.

For midgut and salivary gland dissections, mosquitoes were washed once in 70% ethanol and twice in sterile phosphate-buffered saline (PBS) before dissection. Midguts at 7 days post-exposure (dpe) and salivary glands at 14 dpe were dissected individually in sterile PBS and stored in 150 μl DMEM without additives with sterile glass 0.5-mm microbeads (Next Advance Inc., catalog no. GB-05) at −80°C until extraction and viral RNA quantification via qRT-PCR as described below. Saliva was collected from individual mosquitoes at 14 dpe similarly to what has been described previously (80). Mosquitoes were starved for 4 to 6 hours before saliva collection. At the time of saliva collection, mosquitoes were cold anaesthetized, and legs and wings were removed. At room temperature, the mosquito proboscis was inserted into a 5-μl microcapillary tube (Drummond Scientific, catalog no. 1-000-0050) containing a 1:1 solution of sterile 10% sucrose and FBS. Mosquitoes were allowed to salivate at 27°C with 80% relative humidity for 30 minutes; the contents of the microcapillaries were then expelled into 150 μl DMEM and stored at −80°C until extraction, and *in vitro* assessment for infectious virus was performed as described below. Salivary glands were dissected post-salivation and stored individually in 150 μl DMEM with sterile microbeads at −80°C until extraction and viral RNA quantification via qRT-PCR as described below.

For viral dynamics time course assays, at 0.5 hours post-exposure (hpe), mosquitoes were cold anaesthetized to remove unfed females, and mosquito tissues were dissected for the initial 0.5-hpe time point. For blood meal dissections, bolus-containing midguts were dissected from each of 25 mosquitoes and pooled in 150 μl DMEM. The midguts were gently pestled and vortexed to remove the blood bolus from the midgut tissue, and the samples were subsequently pelleted at 21,000 × *g* for 3 minutes to separate blood and midgut tissue from virus-containing supernatant. Supernatants were collected and stored at −80°C. For midgut dissections, bolus-containing midguts were dissected from each of 25 mosquitoes, washed 4 times in sterile PBS until no blood was visible within the guts, and pooled in 150 μl DMEM. Midgut samples were stored at −80°C. Blood bolus samples were collected at 0.5 hpe and then every 12 hpe until 48 hpe; midgut samples were collected at 0.5 hpe and then every 12 hpe until 96 hpe.

### Viral quantification.

Plaque assay was used to detect infectious virus in mosquito blood bolus samples and to determine the titers of infectious ZIKV in the C6/36 cell culture supernatant in qRT-PCR standard curve infectious range assays (described below). A Vero or BHK-21 cell plaque assay was performed as previously described (79). Briefly, C6/36 supernatants were serially diluted 1:10 6 times in C6/36 cell medium and plated on BHK-21 cell monolayers. Virus titers were assessed in all dilutions with individual plaques to determine average PFU per sample. For blood bolus samples, samples were serially diluted 1:10 6 times in C6/36 cell medium and plated on Vero cell monolayers; virus titers were assessed in all dilutions with individual plaques to determine average PFU per sample. RNA was extracted from remaining samples and quantified by qRT-PCR as described below.

For virus detection by RNA extraction and qRT-PCR, mosquito tissue samples in DMEM containing sterile microbeads were homogenized using a bullet basher (Next Advance Inc.) and pelleted at 21,000 × *g* for 3 minutes to separate mosquito tissues and beads from supernatants containing virus. Mosquito saliva samples were not homogenized but centrifuged briefly to collect sample from the walls of the sample vial. Viral RNA was extracted from supernatants using the QIAamp Viral RNA minikit (Qiagen, catalog no. 52906) per the manufacturer’s instructions with a 60 μl final elution volume. Cell culture samples were lysed in 300 μl RNA lysis buffer, and RNA was extracted using the Quick-RNA Miniprep kit (Zymo Research, catalog no. R1055) according to the manufacturer’s instructions, with a final elution volume of 60 μl. ZIKV RNA titers in samples were quantified via qRT-PCR using the one-step QuantiTect Probe RT-PCR kit (Qiagen, catalog no. 204445), utilizing 5 μl sample RNA in the case of tissue or cell culture samples and 10 μl sample RNA per reaction in the case of mosquito saliva samples, in a 25 μl reaction volume in duplicate wells per the manufacturer’s instructions. Eight-point serial 1:10 dilution standard curves were generated from extracted RNA from ZIKV-Cambodia or ZIKV-Paraiba viral stock, which had been titrated via BHK-21 cell plaque assay. Standard curves and no-template negative controls were run in duplicates on every qRT-PCR plate. The Zika1087/Zika1108FAM/Zika1163c forward/probe/reverse primer combination was utilized as described by Lanciotti et al. to detect all ZIKV genotypes (35).

Samples were run on a Step One Plus real-time PCR system (Applied Biosystems), with 1 cycle of enzyme activation and reverse-transcription reaction (50°C for 30 minutes, 95°C for 15 minutes) and 45 cycles of product amplification (95°C for 15 s, 60°C for 1 minute). ZIKV RNA levels in each sample were calculated as log_10_ (PFU equivalents [p-e]/sample) from mean *C_T_* values via absolute quantification based on the slope and intercept of the standard curve from that plate as well as input sample volume with applicable dilution factors in the case of split samples. Samples that did not amplify in both duplicate wells were assigned a *C_T_* value of 45. Saliva samples that amplified in only 1 of 2 duplicate wells were re-run with 15 μl sample RNA in a 50 μl final reaction volume. Re-run samples that did not produce duplicate *C_T_* values were considered to be non-amplifying and given a *C_T_* value of 45. Tissue samples that produced *C_T_* values in only 1 of 2 duplicate wells were excluded from analysis.

To determine the number of RNA genome copies in the qRT-PCR standard curve generated from extracted RNA from viral stock titers determined from a plaque assay, we calculated genome copies per reaction based on mean *C_T_* values from the standard curve using serially diluted *in vitro* transcribed ZIKV RNA from a full-length infectious viral clone (FLIC) as previously described (81). In brief, the pACYC177 plasmid containing the full-length cDNA genomic sequence from ZIKV-Cambodia (FSS13025, GenBank JN860885) was amplified in chemically competent TOP10 Escherichia coli via Plasmid Plus MaxiPrep (Qiagen, catalog 12965), and RNA was *in vitro* transcribed using the HiScribe T7 Quick RNA Synthesis kit (New England BioLabs, catalog no. E2050S) following linearization with restriction enzyme ClaI. RNA concentration in ng/μl was quantified via a Nanodrop 2000 spectrophotometer (Thermo Scientific) to calculate genome copies per μl. 5 μl of serial dilutions of *in vitro* transcribed RNA was run on multiple qRT-PCR plates as described above, and a linear regression was performed to determine the relationship between log_10_ genome copies per reaction and mean *C_T_* value. This regression equation was subsequently used to calculate the log_10_ (genome copies/reaction) in all standard curves generated from diluted RNA from viral stocks used to calculate sample titers on all qRT-PCR plates in this study.

To determine the range of our qRT-PCR standard curve associated with an infectious quantity of ZIKV, stock ZIKV-Cambodia and ZIKV-Paraiba for which titers were determined by plaque assays were serially diluted 1:10 10 times to create standards (100 μl total volume) with ZIKV PFU levels corresponding to each dilution of the qRT-PCR standard curve and 2 additional dilutions corresponding to less than 1 PFU per sample. Samples were inoculated on 2E05 C6/36 cells in a well of a 24-well cell culture plate. Plates were rocked for 15 minutes at room temperature (RT) and incubated for 45 minutes at 30°C to allow any virions present to infect, after which, 1 ml of C6/36 medium was added to each well. Plates were then incubated for 6 days to allow viral replication and dissemination in any infected wells. Medium was then collected, and RNA was extracted from 140 μl of each sample. Viral RNA levels post-C6/36 amplification were assessed by qRT-PCR, and infectious virus titers were assessed by plaque assay in BHK-21 cells as described above. Three biological replicates were performed. The lowest titer inoculated that always produced infectious virus and viral RNA dissemination in the supernatant above the level of negative controls was considered the lower limit for “positive” samples. The highest titer that never produced infectious virus and viral RNA dissemination in the supernatant was considered the upper limit for “negative” samples. Titers between these limits, producing infectious virus and viral RNA dissemination in supernatants in some but not all biological replicates, were considered “indeterminate” samples. These limits were applied to classify infection status of all mosquito tissue samples assayed in the course of experiments. For samples assessing infectious virus using C6/36 infection *in vitro*, samples were considered infectious if the supernatants of the C6/36 cells post-inoculation were in the positive range of the qRT-PCR standard curve; saliva inoculums producing supernatant titers below this level were considered non-infectious.

To detect potentially low levels of infectious virus in the saliva of ZIKV-fed and ZIKV-injected mosquitoes, ZIKV infectiousness in saliva samples was assessed by *in vitro* infection followed by qRT-PCR. Saliva samples were split, and 50 μl of each sample was diluted 1:1 in C6/36 medium plus 2.5 μg/ml amphotericin B (AMP-B) (Sigma-Aldrich, catalog A2942) and then inoculated onto C6/36 cells which were plated at a density of 2E05 cells/well in a 24-well cell culture plate. Infection was allowed to proceed for 1 hour, at which time, C6/36 medium plus AMP-B was added and the plate was incubated for 6 days at 32°C with 5% CO_2_. RNA was extracted from the remaining 100 μl of sample as described above to determine starting viral titer at the time of saliva collection as well as the amount of virus in the saliva inoculated onto C6/36 cells. After 6 days, the supernatant cell medium was collected. RNA was extracted from 140 μl of supernatant to determine viral RNA concentration post-infection via qRT-PCR as described above. Duplicate negative controls where 50 μl DMEM was diluted 1:1 in C6/36 medium plus AMP-B were included on each plate to determine the background qRT-PCR signal from C6/36 cells/cell medium alone and to detect potential contamination.

### Virus localization.

To assess ZIKV localization in mosquito cell lines by transmission electron microscopy (TEM), A. aegypti Aag2 and *C. tarsalis* ChaoB cells were seeded in 24-well plates at a density between 2E05 and 1E06 cells/well and allowed to attach at 30°C without CO_2_ overnight. Cells were cooled to 4°C, washed once with cold sterile PBS, and subsequently inoculated with ZIKV-Cambodia in Schneider’s *Drosophila* medium with 2% FBS, 1% P/S, at a saturating multiplicity of infection (MOI) of 100 PFU/cell or in medium with no virus for uninfected controls. Cells and virus were incubated at 4°C for 30 minutes to allow for virus synchronization at the cell membrane without internalization. Cells were then moved to 30°C without CO_2_ to permit internalization. At 15 minutes post-exposure (mpe), cells were washed 2 to 3 times in PBS to remove unbound virus and fixed in 2.5% glutaraldehyde, 3 mM MgCl_2_ in 0.1 M sodium cacodylate buffer, pH 7.2, for 1 hour at room temperature. After buffer rinse, samples were post-fixed in 1% osmium tetroxide, 0.8% potassium ferrocyanide in 0.1 M sodium cacodylate for at least 1 hour (no more than 2 hours) on ice in the dark. Samples were then rinsed in 100 mM maleate buffer followed by uranyl acetate (2%) in 100 mM maleate, dehydrated in a graded series of ethanol, and embedded in Eponate 12 (Ted Pella) resin. Samples were polymerized at 37°C for 2 to 3 days followed by 60°C overnight.

Thin sections, 60 to 90 nm, were cut with a diamond knife on a Reichert-Jung Ultracut E ultramicrotome and picked up with 2- by 1-mm Formvar copper slot grids. Grids were stained with 2% uranyl acetate in 50% methanol followed by lead citrate and observed with a Philips CM120 or Hitachi 7600 transmission electron microscope at 80 kV. Representative images for each sample were captured with an AMT charge-coupled-device (CCD) XR80 (8-megapixel camera, side mount AMT XR80, high-resolution high-speed camera). Investigators were blinded to the infection status and cell type of the samples during imaging but unblinded for figure making.

To assess ZIKV localization by TEM in mosquito midgut tissue, Rock and JHB females were exposed to ZIKV via blood meal as previously described. After being allowed to blood feed for 30 minutes on a blood meal containing ZIKV-Cambodia or only C6/36 medium as an uninfected control, mosquitoes were returned to 27°C, 80% relative humidity for 0.5 hpe before being anaesthetized at 4°C. Blood-fed mosquitoes were selected and briefly washed once in cold 70% ethanol and twice in cold sterile PBS. The midguts were dissected in cold sterile PBS without puncturing the blood bolus and fixed and processed as described above, except uranyl acetate staining occurred overnight. Midgut tissues were sectioned between 250 and 500 μm from the posterior end of the gut tissue to target the posterior region of the midgut where virus invasion has been described by previous studies (15, 82–84). Midgut tissue samples were singly stained with 2% uranyl acetate, as when we applied lead citrate staining to midgut tissue samples, we noticed a high frequency of artifacts from the staining bearing close resemblance to the appearance of flaviviruses by TEM. Representative images of each sample were captured as previously described. Due to the low frequency of occurrences of particles resembling flaviviruses, mosquito samples were imaged with knowledge of mosquito species and infection status.

All images were processed, including for diameter measurement of potential viral particles, using ImageJ 1.52p. Histogram matching was applied to all images from each condition. Noise reduction was applied to all images using the “despeckle” function, and contrast was enhanced to allow 0.05% saturated pixels for cell culture samples and 0.1% saturated pixels for midgut samples to improve the clarity of cellular structures in both infected and uninfected samples. The diameter of particles resembling flaviviruses was measured vertically and is recorded in nanometers on each image.

To assess the dynamics of virus invasion in *Aedes* and *Culex* cells, *in vitro* protease treatment assays were used to remove extracellular virus, as has been described (85–88). Aag2 and ChaoB cells were plated in 24-well plates at a density of 1E06 cells per well and allowed to attach at 30°C without CO_2_ overnight. Cells were chilled at 4°C for 15 minutes prior to treatment and exposed to ZIKV-Cambodia at an MOI of 10 in Schneider’s *Drosophila* medium with 2% FBS, 1% P/S. Cells were kept at 4°C for 30 minutes to allow synchronized virus attachment but inhibit virus internalization and rocked gently once every 5 minutes. After 30 minutes, cells were washed three times in cold PBS to remove unbound virus, and then 0.5 ml of cold Schneider’s *Drosophila* medium with 2% FBS, 1% P/S was added per well. The cells were either kept at 4°C to inhibit internalization or moved to 30°C without CO_2_ to permit virus internalization. Treatments occurred at 0.5 hpe for 4°C controls to determine proportions of virus protected from treatment when internalization was inhibited and at 0.5, 3, and 6 hpe for 30°C samples to measure protection from treatment over time at internalization-permissive temperatures. At these time points, cells were returned to 4°C for treatment. Both trypsin and proteinase K protease treatments were used to remove extracellular virus. For proteinase K treatments, at each time point, medium was removed and cells were treated 200 μl of cold proteinase K at 1 mg/ml (Zymo Research, catalog D3001-2-20) for 45 minutes. After treatment, cells were scraped into the proteinase K solution and pelleted at 800 × *g* for 3 minutes. Cells were washed twice with cold PBS plus cOmplete mini protease inhibitor cocktail (Roche Molecular Systems Inc., catalog no. 11836153001) to inhibit further proteinase K activity and then once with cold PBS. Cells were then lysed in 300 μl RNA lysis buffer for RNA extraction and virus quantification. Control cells were processed identically but PBS was used in place of proteinase K. For trypsin assays, cells were washed once with cold PBS to remove residual medium and incubated in cold 0.25% trypsin (Gibco, catalog no. 25200-056) for 5 minutes. After 5 minutes, cells were scraped into the trypsin, collected in 1.5-ml microcentrifuge tubes, and pelleted at 800 × *g* for 3 minutes. Trypsin supernatant was removed and replaced with new trypsin for an additional 5 minute incubation. Cells were pelleted again at 800 to 1,200 × *g* for 5 minutes, trypsin was removed, and cells were lysed in 300 μl RNA lysis buffer (Zymo Research, catalog no. R1055) for RNA extraction and virus quantification. Controls were processed identically, but DMEM with no additives was used in place of trypsin. After RNA extraction, the concentration of RNA in each sample was measured using a NanoDrop 2000 spectrophotometer (Thermo Scientific). After ZIKV quantification in each sample by qRT-PCR, the percentage of virus protected from protease treatment was determined by dividing the amount of virus in the protease treatment well by the amount of virus in the paired control well (in PFU equivalents per well) and multiplying by 100.

### Phylogeny of ZIKV isolates from *Culex* vector competence studies.

Whole-genome sequences from ZIKV isolates that had been tested in published *Culex* mosquito vector competence studies (see Table S1 in the supplemental material) were acquired from NCBI. In cases where the genome accession number for the ZIKV isolate was not included in the publication, all genomes matching that strain identification were included in the analysis. Sequences were aligned using the MUSCLE algorithm with Uclust preprocessing through the Virus Pathogen Resource database (https://www.viprbrc.org). Phylogenic analysis was performed using Unipro Ugene v. 33 using the PhyML maximum likelihood method with a general time reversible (GTR) nucleotide substitution model with 100 bootstrap repetitions (89).

### Statistical analyses.

All statistical analyses were conducted using R Studio version 1.2.5001. For data involving categorical dependent and continuous independent variables (infectious range of qRT-PCR curve, ZIKV blood bolus and midgut time course, and *in vitro* invasion dynamics assays), data were analyzed by ANOVA followed with Tukey’s HSD *post hoc* analysis for comparisons between groups. Models were run with all biological factors, technical factors such as experimental replicates, and relevant interaction terms initially; only non-significant technical factors and interaction terms were subsequently removed from final versions of the model. Non-significant biological factors that were part of the experimental design were included in the final model. Models are specified in figure legends. ANOVA assumptions of homogeneity of variance and normality of distribution were tested using the Levene and Shapiro Wilks’ tests, respectively. Viral titer data (infectious range of qRT-PCR curve and ZIKV blood bolus and midgut time course), were already log-transformed prior to analysis. In cases where the assumption of normality was violated, the statistical analysis was left unaltered, as ANOVA is robust to deviations from this assumption and still the most appropriate test for the experimental design (90, 91). Violations of ANOVA assumptions are included in descriptions of statistical models in figure legends. In the case of the *in vitro* invasion dynamics assay, when the assumption of normality or homogeneity of variance was violated, data were log_10_ transformed for analyses purposes. All figures, with the exception of TEM images, were produced using the ggplot2 package in R Studio, and GNU Image Manipulation Program (GIMP) version 2.10.18 image editing software. TEM figures were made using ImageJ and GIMP.
