# Supplementary material for: Detailed Analyses of Zika Virus Tropism in Culex quinquefasciatus Reveal Systemic Refractoriness
Source: mBio. 2020 Aug 18;11(4):e01765-20. doi: 10.1128/mBio.01765-20 (PMC7439479; doi:10.1128/mBio.01765-20)
Supplement: TABLE S1 [file mBio.01765-20-st001.docx]

**S1 Table. Studies on the vector competence of *Culex* species mosquitoes for ZIKV.** All published studies in which the competence of *Culex* species mosquitoes for ZIKV are listed with information about the mosquito strains, virus isolates, and tissues tested, as well as the infectious assay and qRT-PCR results, when available. In cases where the GenBank accession number of the virus isolate was not included in the publication, the strain name is included in parentheses. Results from studies finding evidence of ZIKV transmission by *Culex* mosquitoes are italicized.

| **Study** | **Species/strain** | **Virus isolate(s)** | **Tissue(s)** | **Infectious assay** | **Infectious assay conclusion** | **qRT-PCR conclusion** | **ZIKV titer in positive samples** |
| --- | --- | --- | --- | --- | --- | --- | --- |
| Aliota et al 2016 [140] | *Cx. pipiens* (Iowa, USA 2002) | KU501215 (PRVABC59) | Bodies, legs, saliva | Vero cell plaque assay | All samples negative | NA | NA |
| Amraoui et al 2016[81] | *Cx. pipiens* (Tabarka, Tunisia 2010)  *Cx. quinquefasciatus* (San Joaquin Valley, CA, USA 1950) | (NC-2014-5132) | Bodies, head, saliva | Vero cell plaque assay | *Cx. pipiens* bodies 1/47 at 7dpe, 5/40 at 21dpe  *Cx. quinquefasciatus* bodies 1/48 at 3dpe, 3/47 at 7dpe, 7/41 at 14dpe, 6/46 at 21dpe;  All *Cx. pipiens* heads and saliva negative;  *Cx. quinquefasciatus* heads 1/41 at 14dpe, 3/40 at 21dpe; All saliva samples negative | NA | Viral loads in bodies between 41 and 56 PFU at 21dpe; viral loads between 10-36 PFU at other time points |
| Boccolini et al 2016[64] | *Cx. Pipiens* (Rome, Italy 2015) | KJ776791.2 (H/PF/2013) | Bodies, legs/wings, saliva | NA | NA | 1/10 *Cx. pipiens* body positive at 3dpe, all negative at subsequent time points, all legs/wings and saliva negative | Viral load in bodies log 0.17 PFU/ml |
| Fernandes et al 2016[67] | Multiple *Cx. quinquefasciatus* strains (Rio de Janeiro, Brazil 2016) | KU926309 (Rio-U1) | Bodies, saliva | Vero cell plaque assay | One *Cx. quinquefasciatus* strain positive in 1/30 bodies with 7 PFU/ml | 2 strains *Cx. quinquefasciatus* positive in 1/30 and 1/16 bodies at 14dpe; saliva not tested | Viral load in bodies between 1,453-1,814 copies/ml |
| Guo et al 2016[60] | *Cx. quinquefasciatus* (Hainan, China 2014) | KU866423 (SZ01/2016/China) | Midgut, salivary glands, ovaries, saliva | NA | NA | *8/10 positive in midgut at 2dpe, 1/10 positive by 8dpe; 9/10 positive in salivary gland at 8dpe; 8/10 saliva samples positive at 8dpe* | *Midgut: log4.94±0.97 copies/ml at 2dpe, log2.98 at 8dpe*  *Salivary gland: log3.92±0.49 copies/ml at 8dpe*  *Saliva: log4.90±0.56 copies/ml at 8dpe* |
| Hall-Mendelin et al 2016[49] | *Cx. annulirostris, Cx. sitiens* (Queensland, Australia)  *Cx. quinquefasciatus* (Brisbane, Australia) | (MR766) | Bodies, legs/wings, saliva | NA | NA | **RT-PCR;** *Cx. auinquefasciatus* positive in 2/30 bodies 14dpe; Additional *Culex* spp negative in all tissues; all *Culex* spp. negative in saliva | NA |
| Huang et al 2016[69] | *Cx. pipiens* (Anderson, CA, USA 2014)  *Cx. pipiens* (Mercer County, NJ, USA 2015)  *Cx. quinquefasciatus* (Vero Beach, FL, USA 2015) | KU501215 (PRVABC59) | Abdomen, head/wings/legs, whole bodiess | Vero cell TCID50 for CPE | All samples negative for cytopathic effects | **RT-PCR**; All samples negative | NA |
| Weger-Lucarelli et al 2016[50] | *Cx. quinquefasciatus* Say (Sebring County, Florida 1988)  *Cx. pipiens* [L.] (Pennsylvania 2002)  *Cx. tarsalis* Coquillett (California 1953) | KU501215 (PRVABC59),  AY632535 (MR766),  KU955591 (SEN/1984/41525-DAK) | Bodies, legs/wings | Vero cell plaque assay | *Cx. quinquefasciatus* positive in 1/48 bodies at 7dpe; Additional *Culex* spp negative in all tissues | NA | NA |
| Dibernardo et al 2017[98] | *Cx. restuans* (Beausejour and Winnipeg, Canada 2016)  *Cx. tarsalis* (Winnipeg, Canada 2016) | KU501215 (PRVABC59),  KF993678 (PLCal_ZV) | Bodies, legs/wings, saliva | NA | NA | All samples negative (Ct <= 38.5) | NA |
| Dodson and Rasgon 2017[141] | *Cx. quinquefasciatus* (Benzon Research colony) | (MR766),  (PRVABC59) | Bodies, legs, saliva | Vero cell plaque assay | All samples negative | NA | NA |
| Fernandes et al 2017[68] | *Cx. quinquefasciatus,* various strains (Recife, Campina Grande, Rio de Janeiro, Brazil | (ZIKVPE243/Recife, NE/2015),  (ZIKVSPH/Sumare, SE/2015)  (ZIKVU1/Rio de Janeiro, SE/2015) | Bodies, heads, saliva | Vero cell plaque assay | *Cx. quinquefasciatus* from Recife: 1/20 bodies positive at 7dpe with ZIKV Rio-U1, head negative | NA | NA |
| Guedes et al 2017[61] | *Cx. quinquefasciatus* (Recife, Brazil 2009  field-collected *Cx. Quinquefasciatus* (Recife Brazil 2016) | KX197192.1 (Brazil/PE243/2015) | Midgut, salivary glands, saliva (via filter paper) | NA | NA | *10/12 positive in midgut at 7dpe, 7/18 positive by 15dpe; 12/12 positive in salivary gland at 7dpe, 5/18 positive by 15dpe* | *Midgut: ~log6 copies/ml 7-15dpe*  *Salivary gland: ~log6.5 copies/ml 7dpe, ~log6 copies/ml 15dpe*  *Saliva: log5-6 copies/ml per card from 9-12dpe* |
| Hart and Roundy et al 2017[66] | *Cx. quinquefasciatus* (Houston, TX, USA)  *Cx. quinquefasciatus* (laboratory colony) | (FSS13025),  (DARKAR41525),  (MEX1-7),  (PRVABC59) | Bodies, legs, saliva | C636 cell focus-forming assay | All samples negative | NA | NA |
| Heitman et al 2017[79] | *Cx, pipiens molestus* (Heidelberg, Germany 2011)  *Cx. pipiens* (Hamburg, Germany 2016)  *Cx. Torrentium* (Hamburg, Germany 2016) | KU870645 (ZIKV_FB-GWUH-2016) | Bodies, saliva | Vero cell assay for CPE, qRT-PCR on supernatant and bodies | No CPE detected in cells inoculated with any *Culex* saliva samples | ZIKV RNA detected in *Cx. pipiens molestus* bodies 7/29 and 12/38, *Cx. pipiens* 3/37 and 0/35, *Cx. torrentium* 4/36 and 0/34 on 14 and 21dpe | RNA copies between log2-4/body |
| Kenney et al 2017[75] | *Cx. quinquefasciatus* (Sebring, FL,USA 1988)  *Cx. pipiens* (Chicago, IL, USA 2010) | (MR766),  (PRVABC59),  (R103451) | Bodies, legs/wings, saliva | Vero cell assay for CPE | MR766: *Cx. quinquefasciatus* bodies 1/95 positive at 14dpe, *Cx. pipiens* bodies 1/20 positive at 14dpe; PRVABC59: *Cx. pipiens* 4/38 bodies positive at 14dpe; R103451: All samples negative; No disseminated infection detected; Saliva samples not tested | NA | 3 PFU/body in *Cx. pipiens* |
| Liu et al 2017[65] | *Cx. quinquefasciagtus* (Guangdong Province, China 1981-1993) | KU820899.2 (ZJ03) | Whole mosquito, midgut, head, salivary glands | NA | NA | 22/138 midguts positive between 4-7dpe, all samples negative after 10dpe; All head, salivary gland samples negative | Log4-5 copies/body between 0-7dpe |
| Main et al 2017[70] | *Cx. quinquefasciatus* (Orange County, CA, USA 2016)  *Cx. tarsalis* (Kern County, CA, USA 2002) | KX601168 (PRVABC59),  KX601167.1 (MA66, P6-740),  KU321639 (BR15, SPH2015) | Bodies, legs/wings, saliva | NA | NA | *Cx. tarsalis* 2/46 positive in body and leg/wing on 14dpe, 6/20 positive in body and 1/20 positive in leg/wing on 21dpe, all saliva samples negative; *Cx. quinquefasciatus* all samples negative (Ct >38) | ~48 copies/body |
| Dodson et al 2018[142] | *Cx. tarsalis* (Yolo County, California, USA 2003) | K501215 (PRVABC59) | Bodies, legs, saliva | Vero cell plaque assay | All samples negative | NA | NA |
| Lourenco de Oliveira et al 2018[143] | *Cx. quinquefasciatus* S-LAB with and without *Wolbachia* | (NC-2014-5132) | Abdomen/thorax, heads, saliva | Vero cell assay for CPE | All samples negative | NA | NA |
| Phumee et al 2019[62] | *Cx. quinquefasciatus* (Nonthaburi Provice, Thailand 2007) | KU681081 (SV0127-14) | Salivary glands, midguts, heads | Immunocytochemistry on tissues post-exposure | *98/112 midguts positive, 62/98 salivary glands positive on 7dpe* | NA | NA |
| Smartt et al 2018[63] | *Cx. quinquefasciatus* (lab colony, 1995) | KU501215.1 (PRVABC59) | Bodies, saliva (via filter paper) | Vero cell plaque assay | *All saliva papers positive* | *Bodies 9/32 positive at 16dpe in first infection, ~18/34 positive in second infection* | *Bodies: log5.85 ± 5.8 pfu equivalents/ml*  *Saliva: log5.6 ± 4.5 pfu equivalents/ml on filters by PCR, log5.02 pfu/ml by plaque assay* |
| Elizondo-Quiroga et al 2019[80] | *Cx. quinquefasciatus* (Guadalajara, Jalisco, Mexico 2016) | Isolated from wild-caught *Cx. quinquefasciatus* | Bodies, heads, saliva | Vero cell assay for CPE | All bodies, heads negative at 14dpe, 1/142 saliva samples positive at 14dpe | 1 saliva sample positive | Saliva titer 502 pfu equivalents/ml by qRT-PCR |
| Hery et al 2019[144] | *Cx. quinquefasciatus* (Guadeloupe, 2018) | KU955592 (Senegal, 1984)  KU647676 (MRS_OPY_Martinique_PaRi_2015)  KX694533 (MYS/P6-740/1966) | Bodies, heads, saliva | TCID50, Vero cell plaque assay | All bodies, heads, saliva negative at 7, 14, and 21dpe | NA | NA |
