# Supplementary material for: Detailed Analyses of Zika Virus Tropism in Culex quinquefasciatus Reveal Systemic Refractoriness
Source: mBio. 2020 Aug 18;11(4):e01765-20. doi: 10.1128/mBio.01765-20 (PMC7439479; doi:10.1128/mBio.01765-20)
Supplement: TABLE S2 [file mBio.01765-20-st002.docx]

**S2 Table.** Summary statistics on Ct values obtained through qRT-PCR analysis of midgut, salivary gland, and saliva samples from Rock, HAI, and JHB strain mosquitoes exposed to ZIKV, including mosquito strain, ZIKV isolate, exposure method, tissue type, days post-exposure (dpe), number of samples analyzed (N), mean Ct value, and 95% confidence interval (ci)

| strain | ZIKV isolate | exposure method | tissue type | dpe | N | mean Ct | ci |
| --- | --- | --- | --- | --- | --- | --- | --- |
| Rock | Cambodia | blood fed | midgut | 7 | 26 | 26.0 | 3.81 |
| HAI | Cambodia | blood fed | midgut | 7 | 38 | 44.1 | 0.909 |
| JHB | Cambodia | blood fed | midgut | 7 | 39 | 43.7 | 0.810 |
| Rock | Cambodia | blood fed | salivary gland | 14 | 40 | 30.1 | 3.04 |
| HAI | Cambodia | blood fed | salivary gland | 14 | 49 | 40.9 | 1.17 |
| JHB | Cambodia | blood fed | salivary gland | 14 | 34 | 43.8 | 1.06 |
| Rock | Cambodia | blood fed | saliva | 14 | 18 | 34.7 | 1.27 |
| HAI | Cambodia | blood fed | saliva | 14 | 24 | 45 | 0 |
| JHB | Cambodia | blood fed | saliva | 14 | 24 | 44.7 | 0.578 |
| Rock | Paraiba | blood fed | midgut | 7 | 31 | 24.3 | 3.43 |
| HAI | Paraiba | blood fed | midgut | 7 | 49 | 45 | 0 |
| JHB | Paraiba | blood fed | midgut | 7 | 47 | 43.7 | 0.985 |
| Rock | Cambodia | injected | salivary gland | 7^a^ | 56 | 23.4 | 0.900 |
| JHB | Cambodia | injected | salivary gland | 7^a^ | 51 | 35.6 | 0.754 |
| Rock | Cambodia | injected | saliva | 7^a^ | 38 | 35.3 | 0.953 |
| JHB | Cambodia | injected | saliva | 7^a^ | 54 | 41.4 | 1.09 |

^a^ – indicates “days post-injection” for mosquitoes injected intrathoracically with ZIKV
